# Protocol for neurogenin-2-mediated induction of human stem cell-derived neural progenitor cells

**DOI:** 10.1016/j.xpro.2024.102878

**Published:** 2024-02-08

**Authors:** Ellen J. Guss, Laila Sathe, Alexander Dai, Tim Derebenskiy, Ana Rodriguez Vega, Kevin Eggan, Michael F. Wells

**Affiliations:** 1 Department of Stem Cell and Regenerative Biology, and Harvard Stem Cell Institute, Harvard University, Cambridge, MA 02138, USA; 2 Department of Human Genetics, David Geffen School of Medicine at the University of California Los Angeles, Los Angeles, CA 90095, USA; 3 Stanley Center for Psychiatric Research, Broad Institute of MIT and Harvard, Cambridge, MA 02142, USA; 4 Molecular Biology Institute, University of California Los Angeles, Los Angeles, CA 90095, USA

**Keywords:** Cell culture, Neuroscience, Stem Cells

## Abstract

Human pluripotent stem cell-derived neural progenitor cells (NPCs) are an essential tool for the study of brain development and developmental disorders such as autism. Here, we present a protocol to generate NPCs rapidly and reproducibly from human stem cells using dual-SMAD inhibition coupled with a brief pulse of mouse neurogenin-2 (Ngn2) overexpression. We detail the 48-h induction scheme deployed to produce these cells—termed stem cell-derived Ngn2-accelerated progenitor cells—followed by steps for expansion, purification, banking, and quality assessment.

For complete details on the use and execution of this protocol, please refer to Wells et al.^1^

## Highlights

• Brief pulse of Ngn2 induces neural progenitor cells from human stem cells
• Guidance on expanding, freezing, and thawing SNaP cells for future use
• Immunostaining-based assays assess cell identity and differentiation potential

## Abstract (editor highlights)

Publisher’s note: Undertaking any experimental protocol requires adherence to local institutional guidelines for laboratory safety and ethics.

## Graphical abstract

**Figure:**
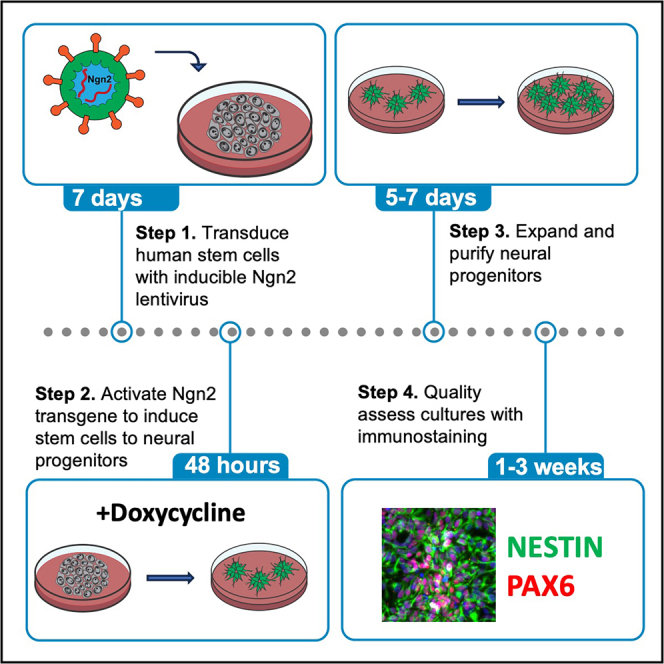

## Before you begin

All procedures involving live cells should be performed under sterile conditions in a Class II biosafety cabinet. All live cell incubations should be cultivated in a humidified incubator at 37°C/5% CO_2_. When feeding, it is important to add media down the sides of the wells rather than directly on top of the cells to prevent detachment.

### Institutional permissions

Human pluripotent stem cell (hPSC) experiments should be conducted in accordance with institutional guidelines and be approved by an institutional review board.

## Key resources table

**Table:** 

REAGENT or RESOURCE	SOURCE	IDENTIFIER
**Antibodies**		

Mouse anti-Nestin (1:1,000)	STEMCELL Technologies	Cat #60091; RRID: AB_2905494
Rabbit anti-PAX6 (1:500)	Cell Signaling Technology	Cat #60433; RRID: AB_2797599
Mouse anti-OCT4 (1:1,000)	STEMCELL Technologies	Cat #60093; RRID: AB_2801346
Rabbit anti-SOX1 (1:1,000)	STEMCELL Technologies	Cat #60095; RRID: AB_2801347
Mouse anti-HuC/D (1:200)	Life Technologies	Cat #A-21271; RRID: AB_221448
Rat anti-CD44 (1:400)	eBioScience	Cat #17-044-82; RRID: AB_469390
Mouse anti-S100B (1:1,000)	Sigma-Aldrich	Cat #S2532; RRID: AB_477499
Donkey anti-mouse Alexa 647 (1:1,000)	Life Technologies	Cat #A-31571; RRID: AB_162542
Donkey anti-rabbit Alexa 555 (1:1,000)	Life Technologies	Cat #A-31572; RRID: AB_162543
DAPI (1:5,000)	Thermo Scientific	Cat #62248

**Chemicals, peptides, and recombinant proteins**		

mTeSR-Plus stem cell media kit	STEMCELL Technologies	Cat #100-0276
MEM media	Thermo Fisher Scientific	Cat #11095
Geltrex basement membrane matrix	Life Technologies	Cat #A1413301
Accutase	Innovative Cell Technologies	Cat #AT104
ROCK inhibitor Y-27632	Stemgent	Cat #04-0012
MEM-NEAA	Life Technologies	Cat #10370088
B27 minus vitamin A	Life Technologies	Cat #12587010
N2 supplement	Life Technologies	Cat #17502048
Recombinant human EGF	STEMCELL Technologies	Cat #78006.1
Recombinant human basic FGF	Thermo Fisher Scientific	Cat #PHG0368
DMEM/F12	Thermo Fisher Scientific	Cat #11320082
PBS with Ca^2+^ and Mg^2+^	Thermo Fisher Scientific	Cat #14040117
GlutaMAX	Thermo Fisher Scientific	Cat #10565018
Glucose	Sigma-Aldrich	Cat #G7021-100G
Doxycycline	Sigma-Aldrich	Cat #D9891
LDN-193189	Stemgent	Cat #04-0074
SB431542	Tocris	Cat #1614
XAV939	Stemgent	Cat #04-00046
Puromycin	Thermo Fisher Scientific	Cat #A1113803
Normocin	InvivoGen	Cat #ant-rn1
Protamine sulfate	MedChemExpress	Cat #HY-107911
Cryostor CS10 freezing media	STEMCELL Technologies	Cat #100-1061
Normal donkey serum	VWR	Cat #102644-006
Triton X-100	Fisher Scientific	Cat #AAA16046AE

**Experimental models: Cell lines**		

Human iPSC: SW7388-1	Nehme et al., 2018	

**Recombinant DNA**		

TetO-Ngn2-Puromycin	Addgene	Cat #52047
FUdeltaGW-rtTA	Addgene	Cat #19780
TetO-EGFP	Addgene	Cat #30130

**Software and algorithms**		

CellProfiler 3.1.9	McQuin et al.^2^	https://cellprofiler.org/previous-releases

**Other**		

Mr. Frosty freezing container	Thermo Fisher Scientific	Cat #5100-0001
Countess III FL automated cell counter	Invitrogen	Cat #AMQAX2000
Countess counting slides	Invitrogen	Cat #C10283

## Materials and equipment

### Stock solutions

• 10 mM Y-27632 ROCK inhibitor: Add 100 mg Y-27632 powder to 31.2 mL sterile water to make a 10 mM stock solution.

Aliquot into 1 mL and store at −80°C until needed.

• 8 mg/mL protamine sulfate: Add 100 mg protamine sulfate powder to 12.5 mL sterile water to make 8 mg/mL stock solution.

Aliquot into 250 μL and store at −80°C until needed.

• 20% glucose: Add 200 g glucose and 800 mL of 1× PBS to 1 L autoclaved glass bottle. Heat to 37°C in a water bath with occasional shaking. Once the glucose is fully dissolved (3–4 h), add 200 mL 1× PBS and sterile filter (0.22 μm) into a new 1 L glass bottle.

Store at 4°C for up to 6 months.

• 20 mg/mL doxycycline: Add 1 g doxycycline powder to 50 mL sterile water.

Aliquot into 1.25 mL and store at −20°C until needed.

• 1 mM LDN-193189: Add 10 mg LDN-193189 powder to 2.25 mL DMSO. Add 100 μL of 10 mM stock solution to 900 μL DMSO to make 1 mM stock solution.

Aliquot into 20 μL and store at −20°C until needed.

• 10 mM SB431542: Add 100 mg SB431542 powder to 23.8 mL DMSO.

Aliquot into 100 μL and store at −20°C until needed.

• 10 mM XAV939: Add 10 mg XAV939 powder to 3.2 mL DMSO in a 15 mL Falcon tube. Invert the tube aggressively until the powder is dissolved.

Aliquot into 20 μL and store at −20°C until needed.

• 10 mg/mL puromycin dihydrochloride: Thaw 1 mL tube of 10 mg/mL puromycin at 20°C–22°C.

Aliquot into 50 μL and store at −20°C until needed.

• 0.1% BSA in D-PBS: Add 133.3 μL 7.5% BSA solution to 9.86 mL D-PBS.

Sterile filter (0.22 μm) and store at 4°C.

• 100 μg/mL human bFGF: Add 500 μL of 0.1% BSA in D-PBS to 50 μg tube of bFGF.

Aliquot into 25 μL and store at −20°C until needed.

• 100 μg/mL human EGF: Add 1 mL of sterile water to 100 μg tube of EGF.

Aliquot into 25 μL and store at −20°C until needed.

• Accutase: Thaw 500 mL bottle of accutase for 12–18 h at 4°C

Aliquot into 10 mL and store at −20°C until needed.

### Preparation of media

**Table:** mTeSR-Plus stem cell media

Reagent	Final concentration	Amount
mTeSR-Plus base	N/A	400 mL
mTeSR-Plus supplement	1×	100 mL
Normocin	500×	1 mL
**Total**		**500 mL**

mTeSR-Plus media should be protected from light and stored at 4°C for no more than 2 days.

***Alternatives****:* This protocol uses mTeSR-Plus, but e8 or standard mTesR could be used as alternatives.

**Table:** SNaP induction base media

Reagent	Final concentration	Amount
DMEM:F12	N/A	49.25 mL
GlutaMAX	1% (1×)	500 μL
20% Glucose	0.3%	750 μL
**Total**		**50 mL**

SNaP induction media should be protected from light and stored at 4°C for no more than 2 days.

**Table:** SNaP induction complete media

Reagent	Final concentration	Amount
SNaP induction base media	N/A	24.7 mL
N2 Supplement	1% (1×)	250 μL
Doxycycline	2 μg/mL	2.5 μL
LDN-193189	200 nM	5 μL
SB431542	10 μM	25 μL
XAV939	2 μM	5 μL
**Total**		**25 mL**

SNaP induction complete media should be protected from light and stored at 4°C for no more than 2 days.

**Table:** SNaP induction selection media

Reagent	Final concentration	Amount
SNaP induction base media	N/A	24.7 mL
N2 Supplement	1% (1×)	250 μL
Doxycycline	2 μg/mL	2.5 μL
LDN-193189	100 nM	2.5 μL
SB431542	5 μM	12.5 μL
XAV939	1 μM	2.5 μL
Puromycin	5 μg/mL	12.5 μL
**Total**		**25 mL**

SNaP induction selection media should be protected from light and stored at 4°C for no more than 2 days.

**Table:** SNaP base media

Reagent	Final concentration	Amount
DMEM:F12	N/A	96 mL
GlutaMAX	2% (2×)	2 mL
Pen/Strep	1% (1×)	1 mL
MEM NEAA	1% (1×)	1 mL
**Total**		**100 mL**

SNaP base media should be protected from light and stored at 4°C for no more than 2 months.

**Table:** SNaP complete media

Reagent	Final concentration	Amount
SNaP base media	N/A	48.5 mL
B27 w/o Vitamin A	2% (1×)	1 mL
N2 supplement	1% (1×)	500 μL
Human EGF	10 ng/mL	5 μL
Human bFGF	10 ng/mL	5 μL
**Total**		**50 mL**

SNaP complete media should be protected from light and stored at 4°C for no more than 2 days.

**Table:** SNaP complete with ROCK inhibitor and puromycin media for additional selection

Reagent	Final concentration	Amount
SNaP complete media	N/A	50 mL
Puromycin	5 μg/mL	25 μL
Y-27632 Rock inhibitor	10 μM	50 μL
**Total**		**50 mL**

SNaP complete media with RI and puromycin should be protected from light and stored at 4°C for no more than 2 days.

## Step-by-step method details

### Preparation of Geltrex-coated plates

**Timing: 7 h (total timing)**

**Timing: 4–6 h (first Geltrex thaw)**

**Timing: 1 h (Geltrex solution preparation and plating)**

Cell culture plates need to be coated with a basement matrix prior to plating cells. Failure to perform this step properly will prevent cells from sticking to the plate and growing.

1. Thaw a tube (5 mL) of LDEV-Free Geltrex for 4–6 h at 4°C until the entire solution is fluid. Once thawed, Geltrex can be stored at 4°C for up to 2 weeks.

***Alternatives:*** This protocol uses Geltrex, but Matrigel could be used in its place.

2. Dilute Geltrex in cold MEM at a ratio of 1:100 to make Geltrex coating solution. This coating solution is stable at 4°C for up to one week.

**CRITICAL:** Geltrex solidifies in 5 min when kept above 15°C. Therefore, keep working vials on ice when in use to prevent premature gelling.

3. Add the Geltrex coating solution to each well of the cell culture-grade plates at 20°C–22°C according to Table 1.

**Table 1:** Media and reagent volumes for different plate formats

Plate format	Surface area	Media feed volume	Geltrex plating solution volume	Accutase volume
6-well	9.6 cm^2^	3 mL	1 mL	750 μL
12-well	3.5 cm^2^	2 mL	600 μL	500 μL
24-well	1.9 cm^2^	1 mL	300 μL	400 μL
48-well	1.1 cm^2^	300 μL	200 μL	300 μL
96-well	0.32 cm^2^	100 μL	100 μL	100 μL
4. Rotate the plates briefly to ensure equal distribution and full coating of the bottom of each well, and incubate for at least 30 min in a 37°C/5% CO_2_ incubator.
5. If using immediately, aspirate the Geltrex coating solution after 30 min and quickly replace with culture media. If not using immediately, the Geltrex-coated plates can be left in the incubator for up to 3 days.

***Note:*** Do not let the Geltrex-coated plate dry if leaving in the incubator for a prolonged period of time or after aspirating the coating solution.

### Thaw and expansion of human pluripotent stem cells (hPSCs)

**Timing: 1–2 weeks**

These steps describe how to thaw and culture hPSCs in 6-well plates in preparation for lentiviral transduction of doxycycline-inducible Ngn2 expression vectors. This feeder-free protocol works for both human induced pluripotent stem cells (hiPSCs) and human embryonic stem cells (hESCs).

6. Thaw hPSCs.
  a. Prepare two Geltrex-coated wells of a 6-well plate.
  b. Label a 15 mL conical tube with the hPSC donor identifier.
  c. Remove the cryogenic vial of hPSCs from the liquid nitrogen storage.
    ***Note:*** If liquid nitrogen tank is more than a 2 min walk from the biosafety cabinet, or if you are thawing multiple lines at the same time, we recommend temporarily placing the cryovials on dry ice to prevent premature thawing.
  d. Quickly thaw cells by gently shaking the vial in a 37°C water bath until only a small frozen pellet of cells is visible. This should take 1–2 min depending on the cell suspension volume.
  e. Make sure the label on the vial is ethanol resistant.
    i. Wipe down the vial with 70% ethanol before transferring into the sterile biosafety cabinet.
    ii. Open the vial and transfer the cell suspension to 15 mL conical tube.
  f. Slowly add 5 mL mTeSR-Plus media dropwise to cell suspension. Gently swirl the tube to ensure complete resuspension and dilution of freezing media.
  g. Centrifuge cells for 5 min at 200 x g to pellet.
  h. Prepare 5 mL mTeSR-Plus with 10 μM Y-27632 Rock Inhibitor (RI).
  i. Aspirate the media from the 15 mL conical tube so that only the cell pellet remains.
    **CRITICAL:** Do not aspirate the cell pellet at this step. If you are concerned about using a vacuum aspirator, use a manual pipet-aid instead.
  j. Add 1 mL of mTeSR-Plus with RI to the cell pellet and gently resuspend without introducing bubbles.
  k. Aspirate the Geltrex coating solution from the 6-well plate and immediately add 2 mL of mTeSR-Plus with RI to each well.
  l. Add 1 million cells dropwise to one well (∼100,000 cells/cm^2^) and 250,000 cells dropwise to another (∼25,000 cells/cm^2^).
    i. If the total number of cells is unknown, instead add 750 μL of cell suspension dropwise to one well and 250 μL to dropwise to the other well.
    ii Gently rock the plate in a T-shape to ensure even distribution of the cells.
      ***Note:*** Plating at multiple densities ensures that at least one well will not be overly dense or sparse during the first 2 days post-thawing. It is best not to passage hPSCs within 2 days of thawing.
  m. Incubate cells for 12–18 h at 37°C/5% CO_2_.
7. Maintain hPSCs.
  a. The day after plating, cells should be fed daily with 2 mL mTeSR-Plus (without RI).
    ***Note:*** mTeSR-Plus allows for a less stringent feeding schedule in which cells can be fed every 48 h. Nevertheless, we feed cells daily during the week and allow for one 48-h feed on weekends.
  b. When cells reach 80%–90% confluency, they should be prepared for passage and expansion.
    ***Note:*** The amount of time it takes to reach this level of confluency depends on the cell line and the initial plating density and survival of the frozen hPSCs. Typically, hPSCs require 3–7 days to reach ∼80%–90% confluency from a thaw.
8. Passage hPSCs.
  a. Thaw a frozen aliquot of Accutase in a 37°C water bath to reach 20°C–22°C.
  b. Prepare two Geltrex-coated wells of a 6-well plate.
  c. Aspirate media from cells and add 750 μL of Accutase to each well. Incubate for 10 min at 37°C/5% CO_2_.
    ***Note:*** At the 5-min mark, briefly remove cells from the incubator and rotate the plate. You should see cells lifting off the plate.
  d. At the 10 min mark, dissociate cells by pipetting up and down 5–10 times. If the cells do not go into suspension, put the plate back into the incubator for an additional 5 min and repeat the dissociation.
  e. Collect the cells into a 15 mL conical tube containing 2 mL mTeSR-Plus so that the ratio of mTeSR-Plus to accutase is at least 2:1.
  f. Centrifuge tube for 5 min at 200 x g to pellet cells.
  g. Prepare 5 mL mTeSR-Plus with 10 μM Y-27632 Rock Inhibitor (RI).
  h. Aspirate the media from the 15 mL conical tube so that only the cell pellet remains.
  i. Add 1 mL of mTeSR-Plus with RI to the cell pellet and gently pipette mix to resuspend.
  j. Aspirate the Geltrex coating solution from the 6-well plate and immediately add 2 mL of mTeSR-Plus with RI to each well.
  k. Add 100 μL of cell suspension dropwise to one well (1:10 passage ratio) and 50 μL dropwise to the other well (1:20 passage ratio). Gently move the plate in a T-shaped motion to ensure even distribution of the cells, pausing between changing directions.
  l. Incubate cells for 12–18 h at 37°C/5% CO_2_.
  m. Feed the cells on the next day by aspirating the spent media and replacing it with mTeSR-Plus media without RI. Cells will be ready for the next passage when they reach 80%–85% confluency 3–5 days later depending on the growth rate of the hPSC line.
    **CRITICAL:** Stem cells should undergo at least one passage prior to transduction to maximize post-transduction survival. One confluent well of a 6-well should be more than enough to generate 1 million cells for lentiviral transduction.

### Lentiviral transduction of hPSCs

**Timing: 7 days (total timing)**

**Timing: 2 h (transduction)**

**Timing: 2–5 days (recovery from transduction)**

**Timing: 1 h (cell banking)**

**Timing: 2 days (lentiviral efficiency test)**

Here, we introduce doxycycline-inducible mouse *Ngn2* (and optionally, GFP) into the genome of human stem cells via lentiviral integration. This protocol has not been optimized for human NGN2.

**CRITICAL:** Safe handling practices and institutional approvals are required for lentiviral transductions.

9. Passage hPSCs for transduction.
  a. Thaw a frozen aliquot of Accutase in a 37°C water bath to reach 20°C–22°C.
  b. Prepare two Geltrex-coated wells of a 12-well plate for Ngn2 lentiviral transduction.
  c. Prepare 5 mL of mTeSR-Plus with 10 μM RI and 8 μg/mL protamine sulfate.
  d. Incubate hPSCs in accutase, dissociate, pellet, and resuspend in 1 mL mTeSR-Plus with RI and protamine.
  e. Count cells using an automated Countess III device (or any other cell counter device)
    i. Add 10 μL of Trypan Blue and 10 μL of cell suspension to a 0.6 mL Eppendorf tube and pipette mix.
    ii. Add 10 μL of cell suspension + Trypan Blue to one side of a Countess slide.
    iii. Place slide in a Countess III automated cell counter, and record the concentration of live cells.
10. hPSC lentiviral transduction.
  a. Remove vials of high-titer TetO-Ngn2-Puromycin and FUdeltaGW-rtTA lentiviruses from the −80°C and place on dry ice. When ready, remove from ice and briefly thaw at 20°C–22°C.
    ***Note:*** We typically purchase lentivirus from Alstem Inc that produces titers between 10^8^ and 10^9^. This protocol is therefore optimized for high-titer viruses. Lower titers may require additional optimization steps.
    ***Note:*** Co-transduction with a tetO-GFP lentivirus is not required but is recommended for test inductions; this enables real-time visualization of cells that were properly transduced and estimations of transduction efficiency.
  b. Add the appropriate volume of lentiviruses to cell suspension of 1 million cells in a 1 mL mTesR-Plus to reach a multiplicity of infection (MOI) of 2 for each lentivirus. Pipette mix 5–10 times.
    ***Note:*** To calculate the volume in microliters of lentivirus needed for MOI = 2 in 1 million cells: Divide 1 million by the viral titer (provided by the commercial lentiviral producer), multiply by 1000 to convert to μL, and multiply that number by 2 (because MOI of 2 is desired). For example, if the viral titer is 1 × 10^9^, then 2 μL should be added to the suspension of 1 million cells [(1 million / 1 × 10^9^) ∗ 1000 ∗ 2)]. This calculation will need to be repeated for each lentivirus, and the appropriate volume of each lentivirus will need to be added to the cell suspension.
    ***Note:*** Lentiviral titers decrease with each freeze-thaw cycle. We recommend making 10 μL aliquots of high-titer virus upon receipt, or purchasing pre-aliquoted lentivirus.
  c. Aspirate the Geltrex coating solution from the 12-well plate and immediately add 500 μL of mTeSR-Plus with RI and protamine sulfate to each well.
  d. Add 500 μL of cell suspension dropwise to each well. Gently rock the plate in a T-shape to ensure even distribution of the cells.
    ***Note:*** The relatively low plating volume (1 mL) ensures adequate concentrations of the lentiviruses.
  e. Centrifuge the plate for 1 h at 1,000 × *g* at 25°C to increase lentiviral contact with the cells (“spinoculation”).
    ***Note:*** We have observed no differences in viability or transduction efficiency when this centrifugation step is performed at 20°C–22°C.
  f. Incubate cells for 12–18 h at 37°C/5% CO_2_.
  g. The next day, add 1 mL mTeSR-Plus media (but no RI) to each well. Incubate for 12–18 h at 37°C/5% CO_2_.
  h. The next day, remove virus-containing media and dispense into a 15 mL conical tube containing 5 mL of 10% bleach. Feed the cells with 2 mL mTeSR-Plus.
    ***Note:*** Cell death is typical within 24–48 h post-transduction. In some cases, this death is concentrated in the center of the well.
  i. Once transduced cells reach 80%–85% confluency (which typically takes 3–5 days depending on the growth rate of the hPSC line and its ability to tolerate lentiviral infection), they can be passaged for freezing, transduction efficiency tests, and/or induction.
11. Banking transduced hPSCs.
  a. Use Accutase to dissociate the cells, then count the cell suspension.
  b. Take 1 million cells and resuspend in 500 μL of Cryostor-10.
  c. Immediately transfer the cell suspension to a labeled 2 mL cryovial.
  d. Place the vial in a Mr. Frosty Freezing container filled with 100%. Place the container in a −80°C freezer.
  e. The next day, move the vial into a liquid nitrogen tank for long-term storage.

**Pause point:** Transduced hPSCs can be stored indefinitely in liquid nitrogen.

**Figure 1:**
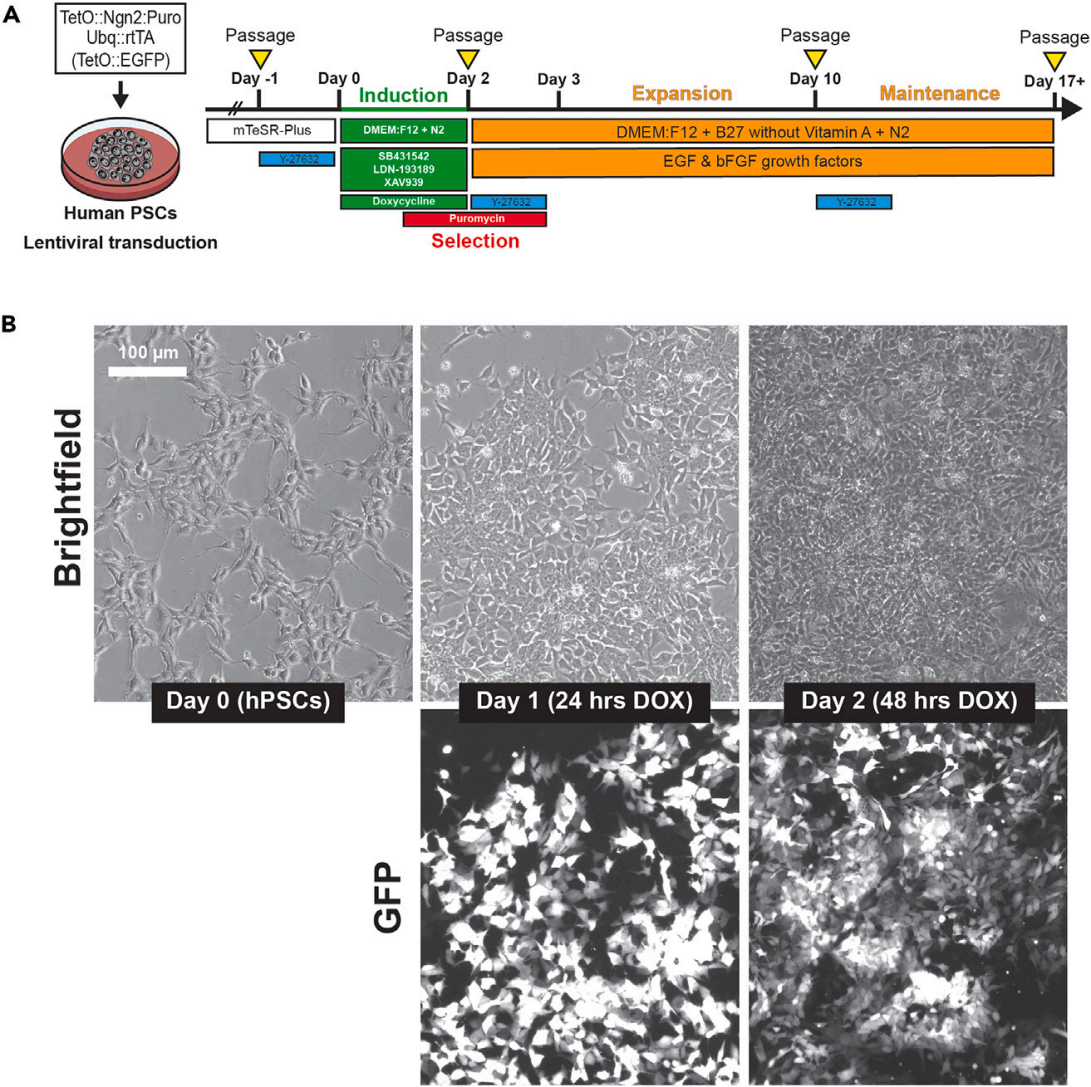
SNaP induction and representative images (A) Schematic describing SNaP induction, selection, and expansion protocol. (B) Bright-field (top) and epifluorescence GFP images (bottom) of cells during the 48 h induction phase. Day 0 stem cells show spindle-like morphology because of the presence of RI. Scale bar = 100 μm.

12. Lentiviral transduction efficiency test.
  a. Use Accutase to dissociate the cells, then count the cell suspension.
  b. Take 75,000 cells and resuspend in 300 μL of mTeSR-Plus with 2 μg/mL Doxycycline.
  c. Plate 100 μL of cell suspension per well of a Geltrex-coated 96-well plate with clear bottoms that are suitable for epifluorescence imaging (3 wells total).
  d. The next day, image the cells using an epifluorescence microscope.
  e. If the GFP lentivirus was included, transduction efficiency can be calculated by estimating the percentage of GFP-positive cells in the culture.
  f. If the GFP lentivirus was not included, transduction efficiency can be calculated by estimating the percentage of cells that show noticeable morphological changes (Figure 1B).

**CRITICAL:** Transduction efficiencies >30% are required for successful NPC induction in the subsequent steps of this protocol. Many factors contribute to differential efficiencies, including the cell line, vendor source of the lentivirus, and the temporal proximity to the lentivirus shelf life. For the SW7388-1 hPSC line, we typically achieve between 75%–95% transduction efficiency. We have observed a broader range of efficiencies across the >100 hPSC lines we have transduced (∼25%–100%).

### Induction of hPSCs to neural progenitors

**Timing: 4 days**

These steps describe how to induce stem cells into neural progenitors through the overexpression of Ngn2. The resulting cells, termed Stem cell-derived Ngn2-accelerated Progenitors (SNaPs), resemble early dorsal telencephalic NPCs at protein, transcriptional, and functional level.^1^

**CRITICAL:** If starting from a thawed vial of transduced hPCS, passage at least one time prior to the start of the induction protocol.

13. Passage and plate 300,000 transduced hPSCs per well into two Geltrex-coated wells of a 12-well plate (∼75,000 cells/cm^2^) in 2 mL mTeSR-Plus media per well.
14. Induce and select SNaPs (Figure 1A).
  a. The next day, aspirate media and feed with 2 mL SNaP induction complete media per well.
  b. Twenty-four hours later, aspirate media and feed with 2 mL SNaP induction selection media per well.
    ***Note:*** At this stage (24 h post-induction), cells should have noticeable morphological changes (Figure 1B). If dox-inducible GFP-expressing lentivirus was included in the transduction, bright green cells should be present. The percentage of cells expressing GFP should be at least 30% to ensure the successful production of an established SNaP line.
  c. Twenty-four hours later (48 h post-induction), dead cells from the puromycin selection should be visible and floating in the media (Figure 1B, top).
    i. If GFP lentivirus was used, a large percentage of remaining cells should be green (Figure 1B, bottom).
      ***Note:*** There may be non-GFP-positive stem cell colonies that remain; these should be eliminated during the following additional selection step.
      ***Note:*** We recommend moving to Step 15 to further purify and expand the SNaP line. However, one could use cells for experiments immediately after the 48 h induction step if: (1) high transduction efficiency (>90%) has been achieved, (2) few stem cell colonies remain, and (3) an adequate number of cells remain for your experiments.
15. First passage and additional selection of SNaPs after 48 h of induction.
  a. Thaw a frozen aliquot of Accutase in a 37°C water bath to reach 20°C–22°C.
  b. Prepare two Geltrex-coated wells of a 24-well plate.
  c. Aspirate media from transduced cells and add 500 μL of Accutase to each well. Incubate for 10 min in 37°C/5% CO_2_ incubator.
  d. After 10 min, dissociate cells by pipetting the cell suspension up and down 5–10 times while applying gentle force to the bottom of the well. If the cells do not go into suspension, put back into the incubator for an additional 5 min and repeat the dissociation.
  e. Move cell suspension to a labeled 15 mL conical tube containing 3 mL SNaP base media and centrifuge for 5 min at 200 × *g* to pellet.
  f. Prepare 4 mL SNaP complete media with 10 μM RI and 5 μg/mL puromycin.
  g. Aspirate the media from the conical tube so that only the pellet remains.
  h. Add 1 mL SNaP complete media with RI and puromycin to the cell pellet and gently resuspend.
  i. Count the cells using a Countess III (or any other counting device).
  j. Aspirate the Geltrex coating solution from the 24-well plate and immediately add 1 mL SNaP complete media with RI and puromycin to each well.
  k. Plate 240,000 cells per well (120,000 cells/cm^2^) by adding the appropriate volume of cell suspension dropwise. Gently rock the plate in a T-shape to ensure even distribution of the cells.
  l. Incubate for 12–16 h at 37°C/5% CO_2_.
  m. No more than 12–16 h later, aspirate the media and feed the cells with 1 mL SNaP complete media.

**CRITICAL:** Do not leave the cells in puromycin-containing media for more than 16 h after passaging. The puromycin resistance cassette is driven by the presence of doxycycline in the media. Doxycycline is removed from our media at 48 h post-induction, as prolonged Ngn2 overexpression forces the cells out of the proliferative state. Therefore, the additional puromycin selection step that takes place after 48-h post-induction relies on the residual protein expression of the puromycin expression cassette. Selection beyond 16 h post-passage will result in excessive death. Failure to include this additional selection step could result in the presence and expansion of stem cell colonies in the SNaP culture.

### SNaP expansion and banking

**Timing: 1–2 weeks**

The induction phase of the SNaP protocol produces cells that resemble dorsal telencephalic NPCs. However, like many hPSC-to-NPC induction protocols, the cells’ morphologies sometimes do not resemble NPCs after the first post-induction passage. The goal of the next set of procedures is to establish a stable line of purified SNaPs that can be maintained for up to 10–15 passages.

16. SNaP expansion.
  a. Continue to feed the cells daily with 1 mL SNaP complete media for one week.
    ***Note:*** In the first 1–2 days post-passaging, it is common to observe cell death, post-mitotic neurons, and flattened cells that do not resemble NPCs. These contaminating cell types, as well as the abnormal morphology of NPCs, tend to resolve themselves over the course of 4–7 days depending on the cell line and the initial transduction efficiency.
  b. When confluency reaches 95%–100% and the cell morphology becomes more uniform within the well, passage and count the cells.
    ***Note:*** Quality control assays (see below) can be performed at this stage of culture development. The SNaP line is considered stable when QC metrics are passed.
  c. Resuspend the cells in 1 mL SNaP complete media with 10 μM RI.
  d. Plate the cells on Geltrex-coated plates in the desired cell culture format at 120,000 cells/cm^2^ by adding the appropriate volume of cell suspension dropwise. Gently rock the plate in a T-shape to ensure even distribution of the cells.
  e. Incubate cells for 12–18 h at 37°C/5% CO_2_.
  f. The next day, feed cells with SNaP complete media.
  g. At this stage, cells should be fed daily and passaged every 7 days.
17. SNaP banking.
  a. Use Accutase to dissociate the cells, then count the cell suspension.
  b. Take 1–2 M cells and resuspend in 500 μL of Cryostor-10.
  c. Immediately transfer the cell suspension to a labeled 2 mL cryovial.
  d. Place the vial in a Mr. Frosty Freezing container filled with 100% isopropanol. Place the container in a −80°C freezer.
  e. The next day, move the vial into a liquid nitrogen tank for long-term storage.

**Pause point:** SNaPs can be stored indefinitely in liquid nitrogen.

18. SNaP thawing.
  a. Prepare two Geltrex-coated wells of a 24-well plate.
  b. Label a 15 mL conical tube with the donor identifier.
  c. Remove the cryogenic vial of SNaPs from the liquid nitrogen storage.
    ***Note:*** If liquid nitrogen tank is more than a 2 min walk from the biosafety cabinet, or if you are thawing multiple lines at the same time, we recommend temporarily placing the cryovials on dry ice to prevent premature thawing.
  d. Quickly thaw cells by gently shaking the vial in a 37°C water bath until only a small frozen pellet of cells is visible. This should take 1–2 min depending on the cell suspension volume.
  e. Make sure the label on the vial is ethanol resistant.
    i. Wipe down the vial with 70% ethanol before transferring into the sterile biosafety cabinet.
    ii. Open the vial and transfer the cell suspension to 15 mL conical tube.
  f. Slowly add 5 mL SNaP base media dropwise to cell suspension. Gently swirl the tube to ensure complete resuspension and dilution of freezing media.
  g. Centrifuge cells for 5 min at 200 × *g* to pellet.
  h. Prepare 5 mL SNaP complete media with 10 μM Y-27632 Rock Inhibitor (RI)
  i. Aspirate the media from the 15 mL conical tube so that only the cell pellet remains.
  j. Add 1 mL of SNaP complete media with RI to the cell pellet and gently resuspend without introducing bubbles.
  k. Aspirate the Geltrex coating solution from the 24-well plate and immediately add 500 μL of SNaP complete media with RI to each well.
  l. Add 500,000 cells dropwise to one well (∼250,000 cells/cm^2^) and 250,000 cells dropwise to another (∼125,000 cells/cm^2^).
    i. If the total number of cells is unknown, instead add 750 μL of cell suspension dropwise to one well and 250 μL to dropwise to the other well.
    ii. Gently rock the plate in a T-shape to ensure even distribution of the cells.
      ***Note:*** Plating at multiple densities ensures that at least one well will not be overly dense or sparse during the first 2 days post-thawing. It is best not to passage SNaPs within 2 days of thawing.
  m. Incubate cells for 12–18 h at 37°C/5% CO_2_.

### SNaP quality control assays

**Timing: 1–3 weeks**

NPCs express canonical protein markers and have the ability to differentiate into neurons and glia. We leverage these molecular and cellular proteins to test the purity of our SNaP cultures and validate the functionality of these NPC models.

19. Marker protein expression.
  a. Use Accutase to dissociate the cells, then count the cell suspension.
  b. Passage 1–2 SNaPs are cultured in 100 μL per well of SNaP complete media with RI at 120,000 cells/cm^2^ on Geltrex-coated 96-well plates with clear bottoms that are suitable for epifluorescence imaging.
  c. The next day, aspirate the media and feed the cells with SNaP complete media without RI.
  d. The next day, fix and stain cells with antibodies directed against NESTIN, PAX6, SOX1, and OCT4 (see immunocytochemistry protocol below).
20. Differentiation assay.
  a. Passage 1–2 SNaPs are cultured at 10,000 cells/cm^2^ in Geltrex-coated 6-well plates in SNaP base media with RI.
  b. After one week, use Accutase to dissociate the cells, then count the cell suspension and plate cells at 10,000 cells/cm^2^ in Geltrex-coated clear-bottomed 96-well plates in SNaP base media without RI.
  c. After one additional week (i.e., after 2 weeks of spontaneous differentiation), fix and stain cells with antibodies directed against HuCD, CD44, and S100ß.
21. Immunocytochemistry protocol.
  a. Aspirate media and gently wash SNaPs with 150 μL 1× PBS with Ca^2+^ and Mg^2+^.
    **CRITICAL:** The cells will detach from the plate if PBS without Ca^2+^ and Mg^2+^ is used.
  b. Immediately remove 1× PBS and add 50 μL of 4% paraformaldehyde. Incubate at 20°C–22°C for 10 min.
  c. Remove the 4% paraformaldehyde and wash cells three times with 150 μL 1× PBS.
    **CRITICAL:** SNaPs tend to lift off the plate if washed too harshly. We recommend leaving a small volume of PBS (10–20 μL) in the wells during each wash.
    **Pause point:** Fixed cells can be stored at 4°C protected from light for 1–2 weeks before permeabilization.
  d. After the final wash, add 50 μL of 0.1% Triton for 15 min to permeabilize cells.
  e. Remove 0.1% Triton and add 50 μL of blocking solution (10% normal donkey serum diluted in 1× PBS) for 1 h at 20°C–22°C. This incubation step should be performed on a plate rocker or shaker.
  f. Prepare the primary antibody in the blocking solution as detailed in Table 2. Remove the blocking solution and add 30–50 μL of primary antibody solution per well.

**Table 2:** Primary antibody dilutions

Antibody	Dilution	Source
Mouse anti-Nestin	1:1000	Stem Cell Technologies (#60091); RRID: AB_2905494
Rabbit anti-PAX6	1:500	Cell Signaling Tech (Cat #60433); RRID: AB_2797599
Mouse anti-OCT4	1:1000	Stem Cell Technologies (#60093); RRID: AB_2801346
Rabbit anti-SOX1	1:1000	Stem Cell Technologies (#60095); RRID: AB_2801347
Mouse anti-HuC/D	1:200	Life Technologies (#A-21271); RRID: AB_221448
Rat anti-CD44	1:400	eBioScience (#17-044-82): RRID: AB_469390
Rb anti-S100ß	1:1000	Sigma Aldrich (#S2532); RRID: AB_477499
  g. Incubate for 12–18 h by shaking gently at 4°C.
  h. The next day, remove the plate from 4°C and allow to equilibrate to 20°C–22°C.
  i. Remove the primary antibody solution and wash three times with 1× PBS.
  j. Prepare the secondary antibody at 1:1000 in blocking solution.
  k. Add 30–50 μL of secondary antibody solution to the cells and incubate for 2–4 h at 20°C–22°C shaking in the dark.
  l. Wash cells once with 1× PBS and add 50 μL of 4′, 6-diamidino-2-phenylindole dihydrochloride (DAPI, 1:5000). Incubate in the dark at 20°C–22°C for 5 min.
  m. Wash cells twice more with 1× PBS prior to imaging.
    **Pause point:** Stained cells can be stored at 4°C protected from light for 1–2 weeks before imaging
  n. For each well of a 96-well plate, we capture 4–8 fluorescent images using the Cytation 5 cell imaging multi-mode reader (Agilent Technologies).
  o. All images are processed using the CellProfiler imaging analysis software^2^ to quantify the percentage of protein marker-positive cells.
22. Quantification of marker-positive cells.
  a. Upload images to CellProfiler using the drag-and-drop feature.
  b. Apply the “IdentifyPrimaryObjects” function to the DAPI images to identify individual cells (Figures 2A and 2B).
    i. The object size should be confined to 20–70 pixels.
    ii. Use the “global” threshold strategy and the “minimum cross-entropy” thresholding method.

**Figure 2:**
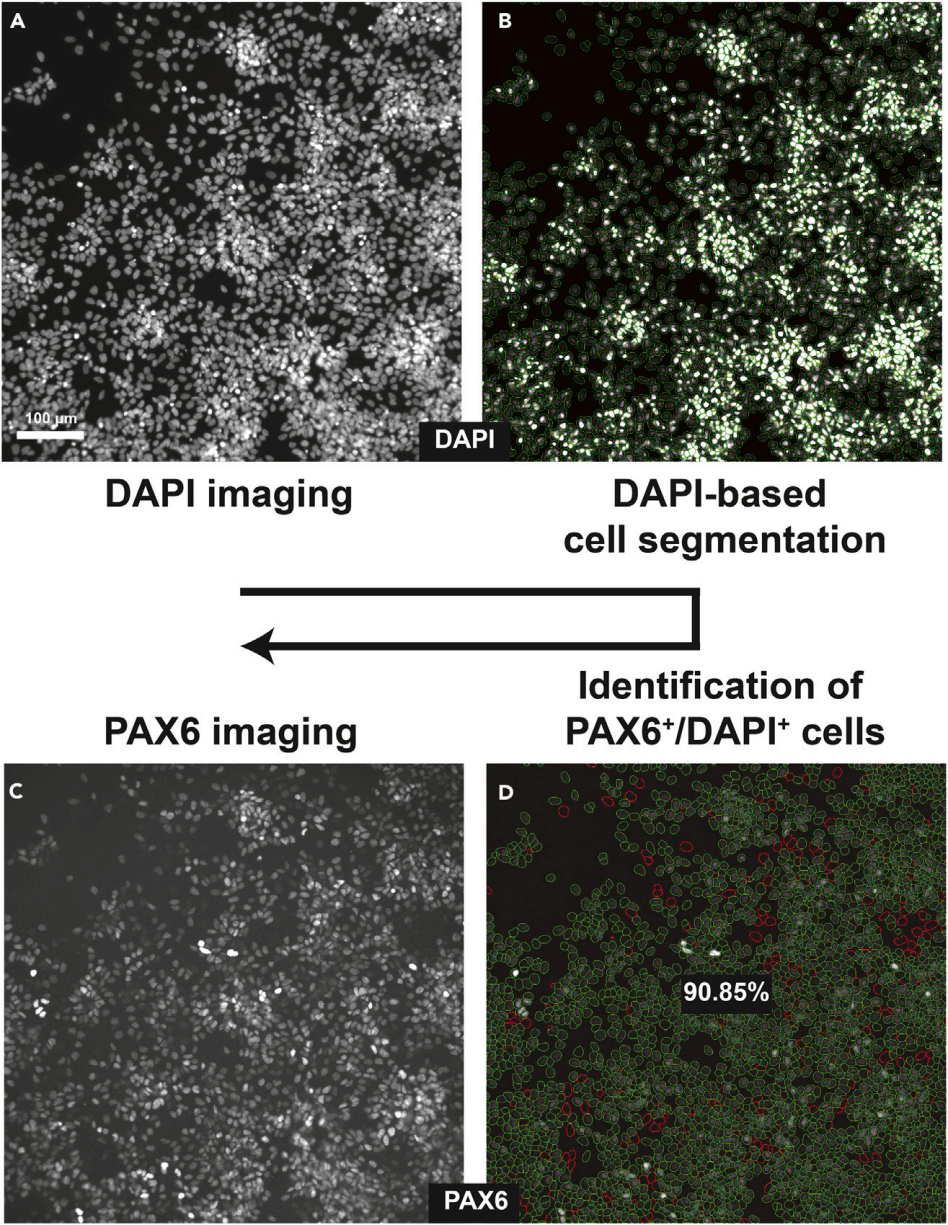
CellProfiler pipeline (A) Epifluorescence image of DAPI^+^ nuclei in Passage 1 SNaPs. (B) Green circles indicate individual cells segmented and identified by CellProfiler. (C) Epifluorescence image of same Passage 1 SNaP culture stained with PAX6. (D) Green circles indicate DAPI^+^ cells that are also PAX6^+^. Red circles indicate DAPI^+^ cells that do not reach PAX6 thresholds and are therefore deemed PAX6^-^. Scale bar = 100 μm.
  c. Repeat this process for the protein marker images (e.g., OCT4, PAX6, etc.) (Figure 2C).
  d. To determine if a marker object overlaps with a DAPI object, first use the “RelateObject” feature in which DAPI is the “parent” and the marker object is the “child.” Repeat this step for each marker.
  e. Then, use the “CalculateMath” function to divide the marker object by the DAPI object.
  f. To generate images depicting object overlap, use the “ConvertObjectsToImage” and “OverlayOutlines” functions (Figure 2D).
  g. Apply the “ExportToSpreadsheet” function to export data that includes readouts of the percentage of marker-positive cells for each image.
  h. For more information on how to use CellProfiler, please refer to publicly-available online tutorials (https://cellprofiler.org/tutorials) and step-by-step protocols.^3^^,^^4^^,^^5^

## Expected outcomes

**Figure 3:**
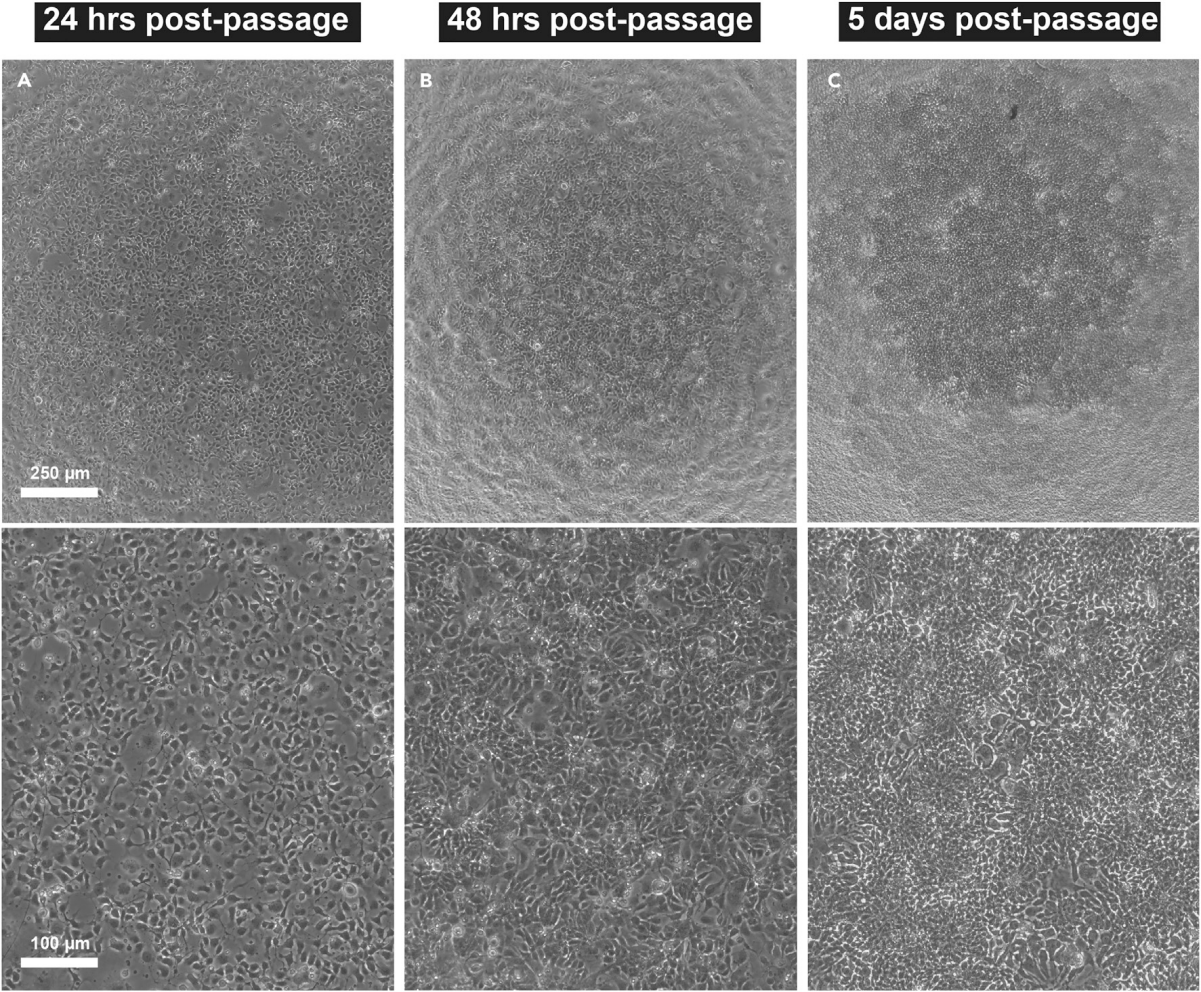
Expansion of SNaP cultures (A–C) Bright-field images of Passage 2 SNaPs (A) the day after passage in the presence of RI, (B) 48 h after passage (24 h after removal of RI), and (C) 5 days post-passage. Top row images captured using a 4× objective (scale bar = 250 μm) and bottom row images captured using a 10× objective (Scale bar = 100 μm).

**Figure 4:**
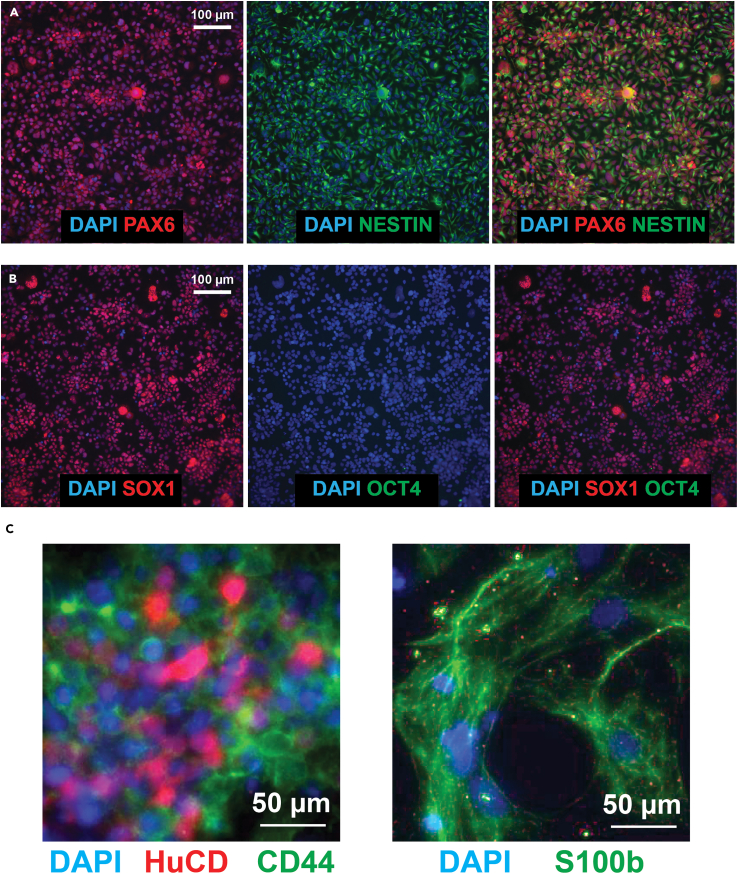
Immunostaining-based SNaP quality assessment (A) Epifluorescence images of PAX6 and NESTIN-stained cells (scale bar = 100 μm). (B) Epifluorescence images of SOX1 and OCT4-stained cells. Scale bar = 100 μm. DAPI used to label nuclei. (C) Epifluorescence images of HuCD^+^ neurons and CD44^+^ glia (left) and S100b^+^ glia after 2–3 weeks of spontaneous differentiation. Scale bar = 50 μm. Images adapted from Wells et al., Cell Stem Cell (2023).

By the end of the second passage (in which the passage after 48-h induction is considered the first passage), there should be a highly pure and homogenous bed of neural progenitors cells (Figure 3). Immunostaining of this culture should yield >80% PAX6^+^/NESTIN^+^/SOX1^+^ and <0.2% OCT4^+^ cells (Figures 4A and 4B). After spontaneous differentiation, both HuC/D^+^ neurons and S100ß^+^/CD44^+^ glial cells should be detected (Figure 4C).

## Limitations

This protocol is dependent on using the exact lentiviral constructs described (e.g., mouse Ngn2). Human NGN2 constructs have not been optimized for SNaP induction. Furthermore, it is imperative to follow the timing instructions for the induction and selection phase, as deviations can result in a failed SNaP culture. The SNaP method has been successful in over 96% of hPSC lines tested; some lines have repeatedly failed induction.^1^

## Troubleshooting

### Problem 1

Low hPSC survival post-transduction.

(Related to Step 10). Some hPSC cell lines are especially sensitive to lentivirus and will die within 48 h after MOI = 2 exposure.

### Potential solution

• Repeat transduction at a lower MOI (∼0.5–1)

### Problem 2

Low transduction efficiency.

(Related to Step 10). Some hPSC cell lines are resistant to lentiviral infection, and investigators will have difficulty achieving adequate transduction efficiencies. We have also observed differences in transduction efficiencies across stem cell maintenance media.

### Potential solution

• Repeat transduction at a higher MOI (∼3).
• If not using mTeSR/mTeSR-Plus, switch to this maintenance media as this has been effective across a range of stem cell lines.

### Problem 3

Low SNaP survival after the 48 h post-induction passage.

(Related to Step 15L-m). This scenario is often the result of failing to remove the puromycin selection media from the culture within 12–18 h after the first passage. The timing of this step is critical.

### Potential solution

• Reduce the duration of puromycin selection after the first passage (Step 15L-m).

### Problem 4

Uneven distribution and low SNaP confluency in the first week after induction.

(Related to Steps 15–16). We have on occasion noticed uneven distribution of SNaPs on cell culture surfaces in which large segments of the plate are missing cells. Typically, we observe these gaps on just one side and/or the center of the wells. In almost all cases, this was the result of media being added too forcefully or directly on top of the cells rather than along the side of the well (N.B. the absence of cells on the left side of the well was common to right-handed feeders; absence on the right side of the well was common to left-handed feeders). SNaPs appear to be most sensitive to feed force in the first couple of days after the 48 h post-induction passage.

### Potential solution

• Always feed cells by adding media along the side of the well, rather than directly from above.
• For especially sensitive cell lines, leave 10% of the media volume in the well prior to feeding with fresh media to reduce cell detachment.

### Problem 5

**Figure 5:**
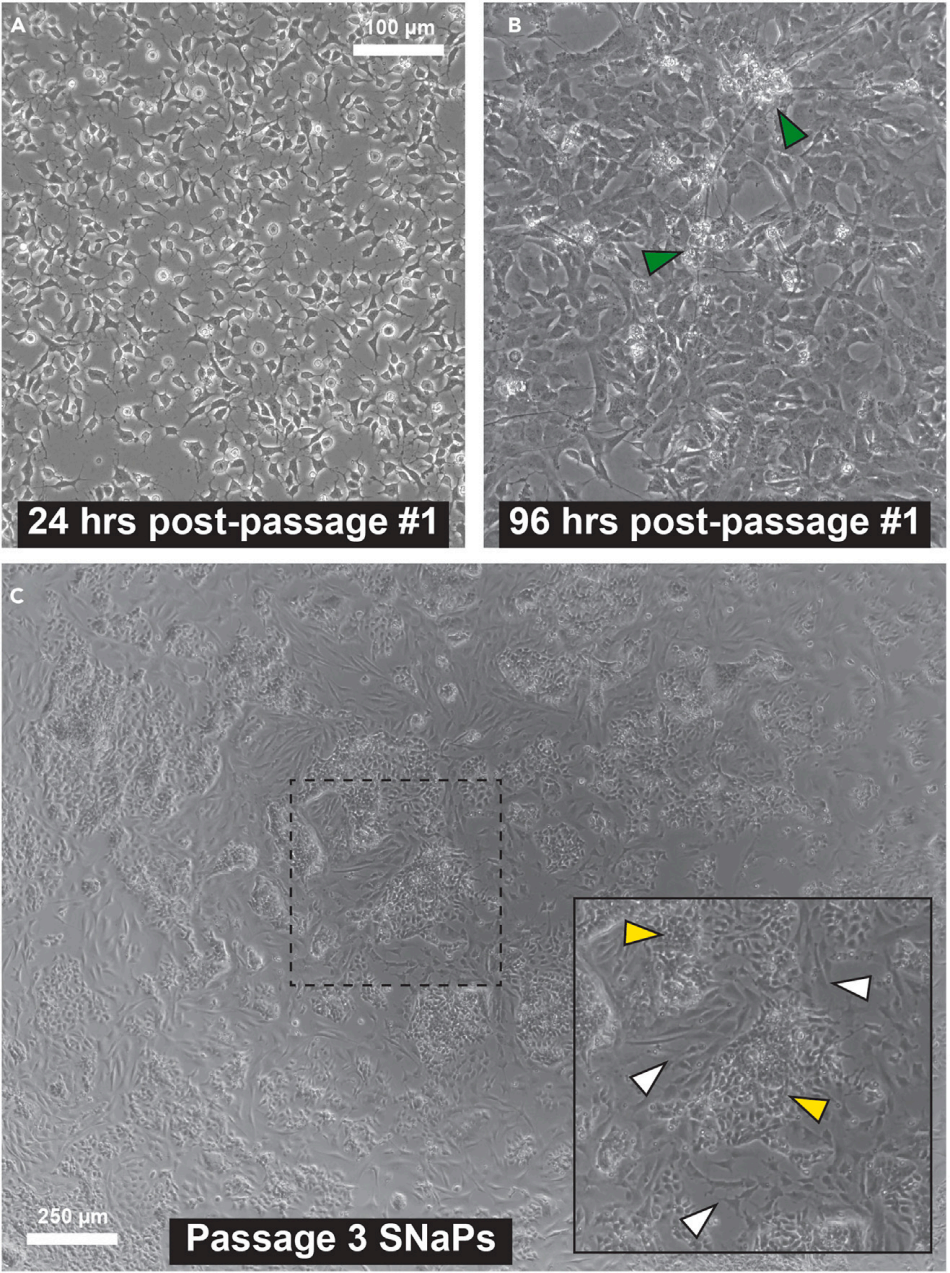
Examples of abnormal morphology (A) One day after the first passage (which takes place 48 h after the start of induction), the cells have a spindle-like morphology as a result of the RI. (B) 2–3 days after first passage, the cells will appear flat and sparse, and neurons will be visible (green arrows). (C) Neural crest cell contamination in Passage 3 SNaP culture. Inset shows SNaP cells (yellow arrows) and neural crest cells (white arrows). Scale bar = 100 μm for A and B; 250 μm for C.

Abnormal morphology in the first week after induction.

(Related to Step 16). In the first 24 h after the 48-h SNaP passage, cells may appear spindle-like (Figure 5A), which is expected and is the result of ROCK pathway inhibition. During the next few days, some SNaP cultures may appear sparse, flat, and/or neuronal in morphology (Figure 5B). This scenario tends to resolve itself with daily feeds no later than 5–7 days after this passage. If the culture is not uniform in morphology within 10 days and/or if the culture does not meet ≥ 75% NPC marker-positivity threshold, we recommend repeating the induction for this line. We have observed a small number of lines that cannot be made into SNaPs; the most common cause of these induction failures is low transduction efficiency. Therefore, if the line fails a second time, we advise taking steps to increase transduction efficiency prior to repeating induction attempts.

### Potential solution

• Increase lentiviral MOI (Step 10) and use mTeSR/mTeSR-Plus to increase transduction efficiency.

### Problem 6

Contaminating cell types in passage 2 SNaP cultures.

SNaP cultures should reach high purity by the end of the first passage, at which point the percentage of cells that remain pluripotent (OCT4^+^) should be less than 0.1%. If, however, this threshold is not met, it is common to observe contaminating cell types in the culture in passages 2 and beyond.

### Potential solution

• Repeat the induction with a prolonged puromycin selection phase during the 48-h post-induction passage (e.g., 18 h instead of 12 h) (Step 15L-m).

### Problem 7

Contaminating cell types in passage 3+ SNaP cultures.

(Related to Step 16). SNaPs should remain proliferative and multipotent for at least 10 passages. Beyond 10 passages, it is common to see low levels of spontaneous differentiation to neural crest, radial glia, neurons, and astrocytes. This differentiation may increase with each passage beyond passage 10. However, we have at times observed SNaP cultures that, despite passing QC and not containing detectable numbers of pluripotent stem cells, show contaminating cells starting at passage 3 (Figure 5C). In almost each case, we were able to attribute this to the inappropriate use of expired EGF/bFGF stocks or repeated feeding of cells with SNaP complete media that was not fresh (i.e., used within 48 h of preparation).

### Potential solution

• Confirm that EGF/bFGF stocks have not exceeded shelf lives.
• Feed the cells daily with fresh SNaP complete media.

## Resource availability

### Lead contact

Further information and requests for resources and reagents should be directed to and will be fulfilled by the lead contact, Dr. Michael F. Wells (mfwells@mednet.ucla.edu).

### Technical contact

Technical questions should be directed to and fulfilled by the technical contact, Dr. Michael F. Wells (mfwells@mednet.ucla.edu).

### Materials availability

This study did not generate new unique reagents.

### Data and code availability

This study did not generate/analyze datasets or code.
